# Composition and Stability of the Oxidative Phosphorylation System in the Halophile Plant *Cakile maritima*

**DOI:** 10.3389/fpls.2019.01010

**Published:** 2019-08-13

**Authors:** Nèjia Farhat, Sarra Hichri, Tatjana Manuela Hildebrandt, Ahmed Debez, Hans-Peter Braun

**Affiliations:** ^1^Laboratory of Extremophile Plants, Center of Biotechnology of Borj Cedria, Hammam-Lif, Tunisia; ^2^Department of Plant Proteomics, Institute of Plant Genetics, Leibniz University Hannover, Hanover, Germany

**Keywords:** halophyte, mitochondria, respiratory chain, oxidative phosphorylation, mitochondrial ATP synthase, Blue native PAGE, *Arabidopsis thaliana*, *Cakile maritima*

## Abstract

Mitochondria play a central role in the energy metabolism of plants. At the same time, they provide energy for plant stress responses. We here report a first view on the mitochondrial Oxidative Phosphorylation (OXPHOS) system of the halophile (salt tolerant) plant *Cakile maritima*. Mitochondria were purified from suspension cultures of *C. maritima* and for comparison of *Arabidopsis thaliana*, a closely related glycophyte (salt sensitive) plant. Mitochondria were treated with digitonin and solubilized protein complexes were analyzed by 2D Blue native/SDS polyacrylamide gel electrophoresis. The OXPHOS systems of the two compared plants exhibit some distinct differences. *C. maritima* mitochondria include a very abundant respiratory supercomplex composed of monomeric complex I and dimeric complex III. At the same time the complexes II and IV are of reduced abundance. The stability of the OXPHOS complexes was investigated by combined salt and temperature treatments of isolated mitochondria. ATP synthase (complex V) is of increased stability in *C. maritima*. Also, the I + III_2_ supercomplex is present in high abundance during stress treatments. These results give insights into the mitochondrial contribution to the plant salt stress response.

## Introduction

Halophile plants have extraordinary competence to live on soils with high contents of salt. This aptitude is based on various physiological properties, like active salt secretion from cells, inner-cellular accumulation of salt in the vacuole and the biosynthesis of compatible osmolytes like proline or glycine betaine (Munns and Gilliham, 2015). In general, increased inner-cellular salt concentrations can cause severe damages, e.g., denaturation of proteins and formation of reactive oxygen species (ROS). However, halophile plants have particular capabilities to cope with these circumstances. For instance, they may have enhanced intra-cellular levels of reductants, such as ascorbate, glutathione or NADPH, which counteract increased ROS formation (Ismail and Horie, 2017). At the same time, enhanced levels of heat stress proteins can stabilize the native structures of proteins.

Plant life in the presence of increased salt levels requires extra energy in the form of ATP (Jacoby et al., 2011, 2018; Bose et al., 2017). ATP is needed for actively secreting salt ions from cells or accumulating salt ions in the vacuole (Nikalje et al., 2018). The corresponding transport processes against the respective concentration gradients are based on proton gradients across the plasma membrane or the tonoplast, which are generated by the act of membrane-bound proton-ATPases (Munns et al., 2016). Furthermore, the biosynthesis of compatible osmolytes, reductants and heat-stress proteins requires additional ATP. In plants, ATP is mainly produced by oxidative phosphorylation (OXPHOS) in the mitochondria and photophosphorylation (PHOTOPHOS) in the chloroplasts. However, PHOTOPHOS only takes place in green cells (it is absent in roots and some organs of flowers) and only operates at daytime. OXPHOS therefore is of outstanding importance for halophyte physiology.

Prerequisite for mitochondrial ATP formation is the respiratory electron transfer chain and the ATP synthase complex (complex V). The respiratory chain is composed of four protein complexes, the NADH dehydrogenase complex (complex I), the succinate dehydrogenase complex (complex II), the cytochrome c reductase complex (complex III), and the cytochrome c oxidase complex (complex IV). Furthermore, cytochrome c, a small monomeric protein, and the lipid ubiquinone are required for the respiratory electron transport. Additionally, in plants and some other groups of organisms, extra enzymes can participate in respiratory electron transport, e.g., the alternative oxidase (AOX) or alternative NADH dehydrogenases (Millar et al., 2011; Schertl and Braun, 2014). As such, the respiratory electron transfer chain is branched, which offers additional physiological functions, but at the same requires some extra levels of regulation.

To our knowledge, the molecular features of the OXPHOS system in halophytes have not been characterized so far. Due to extra energy requirement, we hypothesize that the system should have efficient operation modes. Furthermore, high salt and simultaneously, in some environments, high temperatures might require increased structural stability of the involved protein complexes.

Here, we report the molecular characterization of the OXPHOS system of *Cakile maritima*, a halophile plant. In parallel, the OXPHOS system of the model plant *Arabidopsis thaliana*, a glycophyte (salt sensitive) plant, which also belongs to the Brassicaceae family of angiosperms, is characterized for comparison. For optimal comparability, non-green suspension cell cultures were established for both species and used for parallel mitochondrial isolations. Using two-dimensional Blue native/SDS polyacrylamide gel electrophoresis in combination with differential fluorophore-based labeling of proteins, we here provide insights into the composition and stability of the OXPHOS system of a halophile plant.

## Materials and Methods

### Cell Culture Establishment and Maintenance

#### Establishment of *A. thaliana* and *C. maritima* Callus

Seeds of *A. thaliana* (Columbia 0 ecotype) and *C. maritima* (Raoued ecotype) were sterilized by treatment with 70% ethanol (4 min under shaking) and 6% sodium hypochlorite solution (4 min under shaking). After washing the seeds five times with sterile distilled H_2_O they were plated on solid Murashige and Skoog (MS) medium (Murashige and Skoog, 1962) supplemented with 0.8% agar. Young *A. thaliana* and *C. maritima* plants (age approximately 6 days) grown under sterile conditions were dissected into small pieces with a diameter of about 3 mm. Obtained plant pieces were laid out on solid B5-medium and cultivated in the dark for 2–3 weeks for callus generation.

#### Establishment of *A. thaliana* and *C. maritima* Cell Suspension Culture

*Arabidopsis thaliana* and *C. maritima* cell suspension cultures were established as outlined in May and Leaver (1993): Calli were transferred into 500 mL Erlenmeyer flasks containing 100 mL medium composed of 0.3% (w/v) Gamborg B5 medium supplemented with 3% (w/v) sucrose, 0.01% (w/v) 2,4-D and 0.001% (w/v) kinetin. Cultivation took place at 24°C, at darkness and continuous shaking at 100 rpm. The medium was renewed every 7 days until the generation of approximately 3 g of cell material per Erlenmeyer flask. Afterward, cells were distributed to several flasks. Cell cultures were maintained by transferring about 1.5 g of cells to new medium after 7 days (yielding about 3 g of cells prior to the next round of transferring cells to new medium). Starting material for mitochondria isolations were suspension cells from about 10 Erlenmeyer flasks at the end of a subculturing round (day 7; approximately 30 g of cells in total).

### Mitochondria Isolations

Mitochondria were isolated from *A. thaliana* and *C. maritima* suspension cell cultures as described by Werhahn et al. (2001): The cell cultures (about 30 g of cells per species) were filtered through two layers of Miracloth and homogenized at 4°C in Disruption buffer [450 mM sucrose, 15 mM MOPS, 1.5 mM EGTA, 0.6% (w/v) PVP40, 2% (w/v) BSA, 10 mM sodium ascorbate, 10 mM cysteine, and 0.2 mM PMSF, pH 7.4]. Cells were ground three times using a Waring blender (1 × 15 s at high speed, 2 × 15 s at low speed, 30–60 s intervals in between). The obtained homogenate was then centrifuged twice at 2,700 × *g* for 5 min (organelles in supernatant), once at 8,300 × *g* for 5 min (organelles in supernatant), and once at 17,000 × *g* for 10 min (organelles in pellet). Mitochondria were then resuspended in Wash buffer containing 0.3 M sucrose, 10 mM MOPS, 1 mM EGTA, and 0.2 mM PMSF (pH 7.4) and carefully dispersed using two strokes in a Teflon homogenizer. The resulting suspension was transferred on top of Percoll gradients [3 gradients per species; 18, 23, and 40% Percoll prepared in Gradient buffer (0.3 M sucrose, 10 mM MOPS, pH 7.4)]. After ultracentrifugation for 90 min at 70,000 × *g*, the mitochondria were collected from the 23/40% interphase of the gradients. Percoll was removed by three rounds of resuspending the mitochondria in Resuspension buffer (0.4 mM mannitol, 1 mM EGTA, 10 mM Tricine, and 0.2 mM PMSF, pH 7.2)/re-collecting them by centrifugation at 14,500 × *g* for 10 min. Final mitochondrial pellets were resuspended in Resuspension buffer (weight corresponding to 10× the weight of the mitochondrial pellet). Organelle suspensions were finally divided into aliquots of 100 μL and directly used for gel electrophoresis (see below) or shock frozen and stored at −80°C.

### Mitochondria Solubilization and 2D BN/SDS–PAGE

Isolated mitochondria of *A. thaliana* or *C. maritima* (aliquots of 100 μL corresponding to 10 mg mitochondria) were sedimented by centrifugation for 10 min at 14,300 × *g*. Resulting pellets were re-suspended in 100 μL of Digitonin solubilization buffer, pH 7.4 [30 mM HEPES, 150 mM potassium acetate, 10% (v/v) glycerol, and 5% (w/v) digitonin]. For salt-treatment, mitochondria were resuspended in Digitonin solubilization buffer (see above) supplemented with 300 mM NaCl. Suspensions were kept on ice for 20 min or incubated for 2 or 5 min at 50°C. After incubation on ice or at 50°C, insoluble material was removed from the suspensions by centrifugation for 10 min at full speed and 4°C. Obtained supernatants were supplemented with 5 μL of Coomassie-blue solution [750 mM aminocaproic acid, 5% (w/v) Coomassie-blue 250 G].

Fractions were directly loaded onto a Blue native (BN) gel. 2D BN/SDS–PAGE was performed as outlined by Wittig et al. (2006). Blue native separation of protein complexes was carried out in gradient gels of 4.5–16% (w/v) polyacrylamide. SDS–PAGE for second gel dimension was carried out in a separation gel [constant polyacrylamide concentration of 16.5% (w/v)], which was overlaid with a 10% (w/v) spacer gel. After completion of the electrophoretic runs, gels were fixed for 2 h [fixing solution: 15% (v/v) ethanol, 10% (v/v) acetic acid] and stained according to the Coomassie-blue colloidal protocol [staining solution: 5% (w/v) Coomassie-blue, 2% ortho phosphoric acid, and 10% (w/v) ammonia sulfate] as described by Neuhoff et al. (1985, 1990).

All comparative proteome analyses were based on at least four independent experiments (biological controls) and data evaluation using the Delta 2D software package, version 4.3 (Decodon, Greifswald, Germany) according to Berth et al. (2007) and Lorenz et al. (2014).

### Fluorescence Differential Gel Electrophoresis (DIGE)

Fluorescent differential gel electrophoresis (DIGE) in combination with 2D Blue native/SDS–PAGE was carried out as outlined in Heinemeyer et al. (2009). CyDyes were obtained from GE Healthcare (Munich, Germany). 100 μL of the mitochondrial solutions prepared from *A. thaliana* and *C. maritima* (see above) were solubilized using Digitonin solubilization buffer (see above) and subsequently incubated with either Cy5 or Cy3 for 10 min. The reaction was stopped by addition of 1 μL Lysine solution (10 mM lysine). The two fractions were finally mixed, supplemented with Coomassie-blue buffer and proteins were separated by 2D Blue native/SDS–PAGE as given above. For details see Heinemeyer et al. (2009).

## Results

Suspension cell cultures are an ideal system for investigating house-keeping functions of organisms because they can be maintained at very defined conditions. Suspension cell cultures for *A. thaliana* were first established 25 years ago (May and Leaver, 1993). Their physiological properties have been carefully investigated (Davy de Virville et al., 1994, 1998). Meanwhile, numerous studies have been carried out for investigating the basic functions of cells in *A. thaliana* (e.g., Kruft et al., 2001; Lee et al., 2008). Successful establishment of a suspension cell culture for *C. maritima* was only reported 5 years ago (Ben Hamed et al., 2014). Halophyte behavior of the cells is maintained in the cell culture (Ben Hamed-Laouti et al., 2016). If compared to a suspension cell culture from *A. thaliana*, the cell death rate upon treatment with 400 mM NaCl was much reduced. Indeed, *C. maritima* suspension cells have remarkable properties for NaCl exclusion. At the same time, they have increased inner-cellular ascorbate levels (Ben Hamed-Laouti et al., 2016). We conclude that cell cultures are a suitable starting material for investigating protein complex stabilities in *A. thaliana* and *C. maritima*.

**FIGURE 1:**
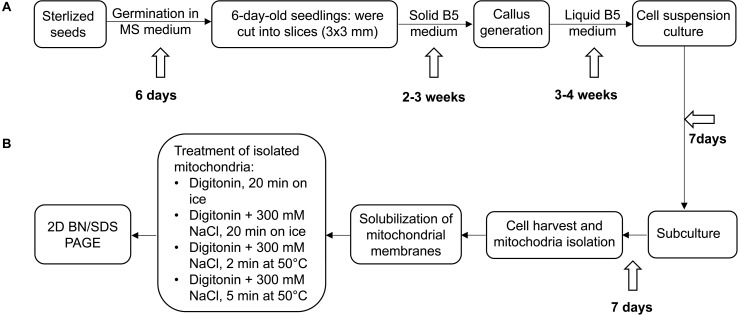
Experimental outline. **(A)** Establishment of *Arabidopsis thaliana* and *Cakile maritima* cell suspension cultures, **(B)** further processing of cell cultures, mitochondria isolation and solubilization. For details see Materials and methods section.

For investigating the OXPHOS system, suspension cell cultures for *A. thaliana* and *C. maritima* were established simultaneously as outlined in Figure 1. After subculturing for 7 days, about 30 g cells per species were used as starting material for parallel mitochondrial isolations by differential centrifugation and Percoll density gradient centrifugation. The protocol used for preparing mitochondria from *A. thaliana* suspension cell cultures has been evaluated previously and shown to generate very pure organelle fractions (purity > 95%; Senkler et al., 2017). The yield of a typical organelle preparation was about 150 mg mitochondria (pellet weight) per 30 g of *A. thaliana* or *C. maritima* cells. Mitochondrial fractions of both species were divided into aliquots corresponding to about 1 mg mitochondrial protein, shock-frozen in liquid nitrogen and stored at −80°C.

### The OXPHOS Complexes in *A. thaliana* and *C. maritima*

**FIGURE 2:**
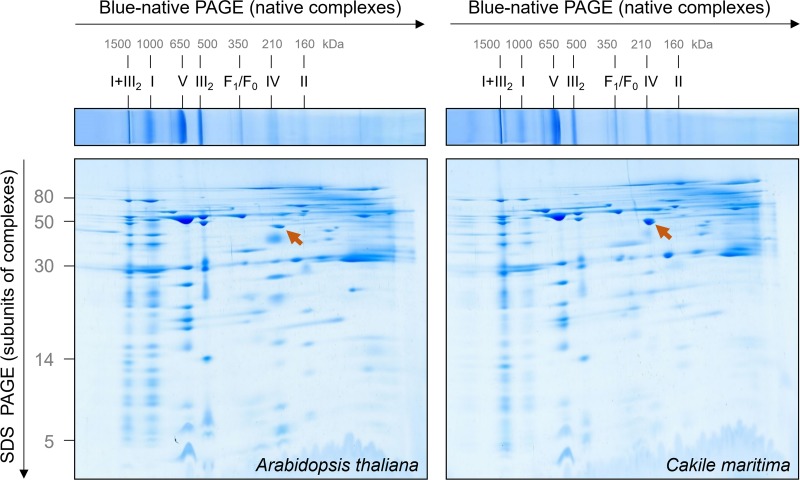
Comparative analysis of the mitochondrial proteomes of *Arabidopsis thaliana* and *Cakile maritima* by Blue native/SDS–PAGE. Mitochondria purified from cell suspension cultures were solubilized by digitonin. Subsequently, protein fractions were supplemented with Coomassie-blue and separated by 2D Blue native/SDS–PAGE. After the second gel dimension, gels were Coomassie stained. Molecular masses of standard protein complexes are given above, molecular masses of monomeric proteins to the left of the 2D gel (in kDa). Identities of the protein complexes are given above the BN gel. Designations: I + III_2_, supercomplex composed of complex I and dimeric complex III; I, complex I; V, complex V (ATP synthase); III_2_, dimeric complex III; F_1_, F_1_ part of ATP synthase; F_0_, F_0_ part of ATP synthase; IV, complex IV; II, complex II. The arrow points to glutamate dehydrogenase.

The mitochondrial OXPHOS system can be nicely characterized by Blue native gel electrophoresis (Schägger and von Jagow, 1991). Mitochondrial membranes are carefully dissolved by a mild non-ionic detergent and solubilized protein complexes incubated with a Coomassie-blue solution for careful introducing negative charge into protein complexes. Subsequently, protein complexes are separated by electrophoresis in polyacrylamide gradient gels. The native gel dimension can be combined with SDS–PAGE in orthogonal direction to separate subunits of protein complexes (Schägger and von Jagow, 1991). Mitochondrial fractions from *A. thaliana* and *C. maritima* were solubilized by digitonin (5 mg/mg mitochondrial protein) and supplemented with Coomassie-blue solution. The result of a typical 2D Blue native/SDS–PAGE analysis is shown in Figure 2.

The OXPHOS system of *A. thaliana* mitochondria has been extensively characterized by 2D Blue native/SDS–PAGE in combination with mass spectrometry (Klodmann et al., 2011). A GelMap of the Arabidopsis mitochondrial proteome from cell culture is presented at the GelMap portal^1^. This data background allows evaluating the 2D gels shown in Figure 2. Overall, the OXPHOS systems in *C. maritima* and *A. thaliana* are highly similar, which can be expected because both species are from the same family. However, some features clearly differ: (i) Nearly all complex I forms a respiratory supercomplex with dimeric complex III in *C. maritima*; at identical conditions, only about 50% of complex I is associated with dimeric complex III in *A. thaliana*. (ii) The complexes II and IV are of decreased abundance in *C. maritima*. (iii) Besides differences related to the OXPHOS system, *C. maritima* contains high amounts of the glutamate dehydrogenase complex.

### OXPHOS Subunits in *A. thaliana* and *C. maritima*

**FIGURE 3:**
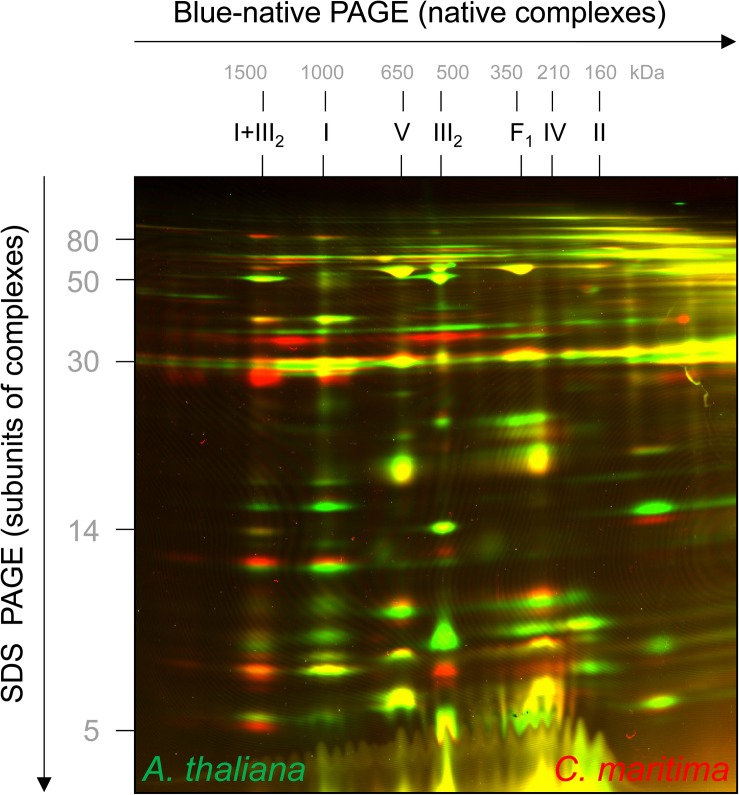
2D fluorescence differential gel electrophoresis analysis of mixed mitochondrial fractions of *A. thaliana* and *C. maritima*. Proteins of *A. thaliana* and *C. maritima* mitochondrial fractions were labeled with different CyDyes (Cy3 for *A. thaliana* and Cy5 for *C. maritima*) and separated by 2D Blue native/SDS–PAGE. Visualization was carried out by laser scanning at the respective wavelengths using the Typhoon laser scanner (GE Healthcare, Munich, Germany). Identities of the resolved protein complexes are given above the 2D gel (for designations see legend of Figure 2). On the resulting overlay image, proteins of *A. thaliana* are seen in green and those of *C. maritima* in red. If proteins exactly overlap, they are seen in yellow.

*Cakile maritima* and *A. thaliana* are closely related organisms, but the amino acid sequences of their subunits forming part of OXPHOS complexes differ slightly (precise information will become clear upon knowledge of the genome sequence of *C. maritima*). To visually compare molecular masses of subunits of the two species, mitochondrial fractions were differentially labeled with CyDye fluorophores, mixed, and co-electrophoresed by 2D Blue native/SDS–PAGE. On the resulting overly image, *C. maritima* proteins are red and *A. thaliana* proteins green (Figure 3). If proteins exactly match in size and abundance, the spots on the resulting 2D gel are visible in yellow. Several of the subunits of the complexes III and V are yellow, indicating similar subunit abundances and sizes. However, distinct subunits are visible as pairs of spots in red + green, indicating differences in subunit size between the two compared species. Subunits of the I + III_2_ supercomplex are more abundant in *C. maritima* (red) and subunits of monomeric complex I, complex II and complex IV in *A. thaliana* (green), supporting our results obtained by Coomassie-staining of the 2D Blue native/SDS gels shown in Figure 2.

### Controlled Destabilization of OXPHOS Complexes of *A. thaliana* and *C. maritima* by Salt and Temperature

**FIGURE 4:**
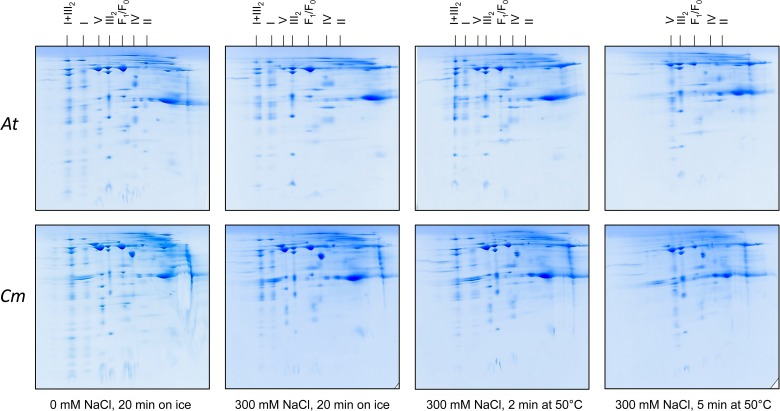
Salt and heat stability of the mitochondrial OXPHOS complexes from *A. thaliana* (*At*) and *C. maritima* (*Cm*). Isolated mitochondria were treated as indicated below the 2D gels. Subsequently, mitochondria were solubilized by digitonin and mitochondrial proteins separated by 2D Blue native/SDS–PAGE. Identities of the resolved protein complexes are given above the 2D gels. For designations see legend of Figure 2.

We used the Raoued ecotype of *C. maritima* and the Col-O ecotype of *A. thaliana* for our investigations. Raoued is from the Mediterranean coast of Tunisia (20 km to the north of Tunis) while Col-O probably originated from central Germany (see Nordborg et al., 2005 and Somssich, 2018 for discussion). The two ecotypes are adapted to differing environments. Raoued not only is exposed to salty soils but also to increased average temperatures when compared to the Col-O ecotype of *A. thaliana*. What are the molecular adaptations of the two ecotypes compared in our study? We hypothesize that high salt and simultaneously high temperatures may have promoted evolution of protein complexes of increased stability in *C. maritima* ecotype Raoued. This was tested by directly exposing mitochondrial protein fractions of *A. thaliana* and *C. maritima* to salt and temperature followed by 2D Blue native/SDS–PAGE evaluation. Mitochondrial protein fractions of *A. thaliana* and *C. maritima* (1 mg of mitochondrial protein in 100 μL of Digitonin solubilization buffer; see Materials and methods) were treated with different NaCl concentrations and temperatures for defined periods of time. Conditions were systematically optimized for defining boundary values with respect to protein complex stability (data not shown). The following treatments were finally used: (i) 0 mM NaCl at 0°C for 20 min (= control), (ii) 300 mM NaCl at 0°C for 20 min, (iii) 300 mM NaCl at 50°C for 2 min, afterward 0°C for 18 min, (iv) 300 mM NaCl at 50°C for 15 min, afterward 0°C for 15 min. All samples were analyzed by 2D Blue native/SDS–PAGE and proteins were visualized by Coomassie-blue staining (Figure 4).

In *A. thaliana*, ATP synthase (complex V) abundance on the 2D gels significantly drops in fractions treated with 300 mM NaCl (Figure 4). Also, complex II of *A. thaliana* clearly is destabilized by 300 mM NaCl (Figure 4). Complexes I and IV as well as the I + III_2_ supercomplex of *A. thaliana* are stable in 300 mM salt, even if treated for 2 min at 50°C. In contrast, all three complexes completely disappear on the 2D gels upon treatment for 5 min at 50°C (Figure 4). Dimeric complex III proved to be the most stable OXPHOS complex in *A. thaliana*. It is not even affected at 300 mM NaCl and treatment at 50°C for 5 min (Figure 4).

In *C. maritima*, salt and temperature treatments led to very similar effects. However, some differences with respect to *A. thaliana* were visible: (i) Complex V is more stable in *C. maritima* upon salt treatment. (ii) Amount of dimeric complex III even increases upon combined salt and heat treatment. This result probably reflects dissociation of the I + III_2_ supercomplex (which is more abundant in *C. maritima*) into monomeric complex I and dimeric complex III. (iii) Besides the OXPHOS complexes, the glutamate dehydrogenase complex is of increased abundance in *C. maritima* mitochondria. It is clearly affected by salt and temperature. However, a small percentage of this complex was stable even at the harshest conditions. All experiments illustrated in Figure 4 were repeated several times (three biological controls and several technical controls) and quantitatively evaluated using the Delta 2D software package (Supplementary Figures 1, 3). A statistical evaluation of the results has been exemplarily carried out for the ATP synthase complex (Supplementary Figure 2).

## Discussion

This study is dedicated to the OXPHOS system of *C. maritima*. As expected, the OXPHOS system resembles that of the closely related model plant *A. thaliana*, which has been characterized extensively (Eubel et al., 2003; Klodmann et al., 2011; Senkler et al., 2017). In Arabidopsis, complex I consists of about 49 subunits, complex II of 8 subunits, dimeric complex III of 2 × 10 subunits, complex IV of about 13 and ATP synthase of about 15 subunits. In *C. maritima*, we could not detect any differences with respect to subunit numbers of the five OXPHOS complexes. However, several OXPHOS subunits have slightly varying molecular masses (Figure 3). Furthermore, the stoichiometry of the OXPHOS complexes differs between *A. thaliana* and *C. maritima* (Figure 2). In *C. maritima*, the I + III_2_ supercomplex is more abundant (Figure 2). This may promote efficient electron transfer from complex I to complex III. Furthermore, it also may have a positive effect on the stability of the monomeric complexes. However, the precise physiological role of respiratory supercomplexes is still a matter of debate (see Hirst, 2018 for discussion). In contrast, complexes II and IV seem to be of reduced abundance in *C. maritima*. This could affect the capacity for electron insertion into the respiratory chain (ETC)/reduction of oxygen to water by the ETC. The latter effect could be compensated by AOX (this enzyme is difficult to detect on BN/SDS gels). Finally, glutamate dehydrogenase is quite prominent in *C. maritima*. Halophile plants usually have much increased capacities for proline biosynthesis during salt stress as well as proline catabolism upon salt stress release. Glutamate dehydrogenase is involved in the mitochondrial proline degradation pathway.

Salt and temperature might affect protein and protein complex stability. Indeed, it is known that species living in very hot environments have protein complexes of high stability (Graziano and Merlino, 2014). For this reason, structural analyses of proteins and protein complexes using x-ray crystallography or single particle cryo electron microscopy often is performed with protein fractions isolated from thermophilic bacteria. Also, prerequisite of the polymerase chain reaction (PCR) is a heat-stable DNA polymerase like present is thermophilic bacteria. Due to its natural environment, we hypothezised hat *C. maritima* may have more stable OXPHOS complexes than the glycophyte plant *A. thaliana*. However, the stability of the OXPHOS complexes turned out to be similar. Indeed, the natural environments of these ecotypes do not differ drastically. At the same time, some differences were observed. The ATP synthase complex (complex V) was slightly more stable in *C. maritima*. This complex is in the very center of mitochondrial ATP production. Furthermore, the I + III_2_ supercomplex is very abundant in *C. maritima*. Only at the harshest treatment condition (300 mM NaCl and 50°C for 5 min), this supercomplex dissociates. As a result, the amount of dimeric complex III increases, while the complex I monomer is degraded. Physiological experiments using isolated mitochondria from *A. thaliana* and *C. maritima* should be employed next to further compare mitochondrial functions in these two species in the presence and absence of stress factors like salt. This should give further insights into the mitochondrial contribution to the salt stress response in plants.

## Data Availability

Available data are presented in the manuscript and the Supplementary Material.

## Author Contributions

NF performed all the experiments. NF, SH, and TMH carried out the Delta2D evaluations. H-PB and AD designed and supervised the project. H-PB and NF wrote the manuscript.

## Conflict of Interest Statement

The authors declare that the research was conducted in the absence of any commercial or financial relationships that could be construed as a potential conflict of interest.

## Footnotes

1 https://www.gelmap.de/1227
