# Supplementary figures and images for: Composition and Stability of the Oxidative Phosphorylation System in the Halophile Plant Cakile maritima
Source: Front Plant Sci. 2019 Aug 13;10:1010. doi: 10.3389/fpls.2019.01010 (PMC6700300; doi:10.3389/fpls.2019.01010)

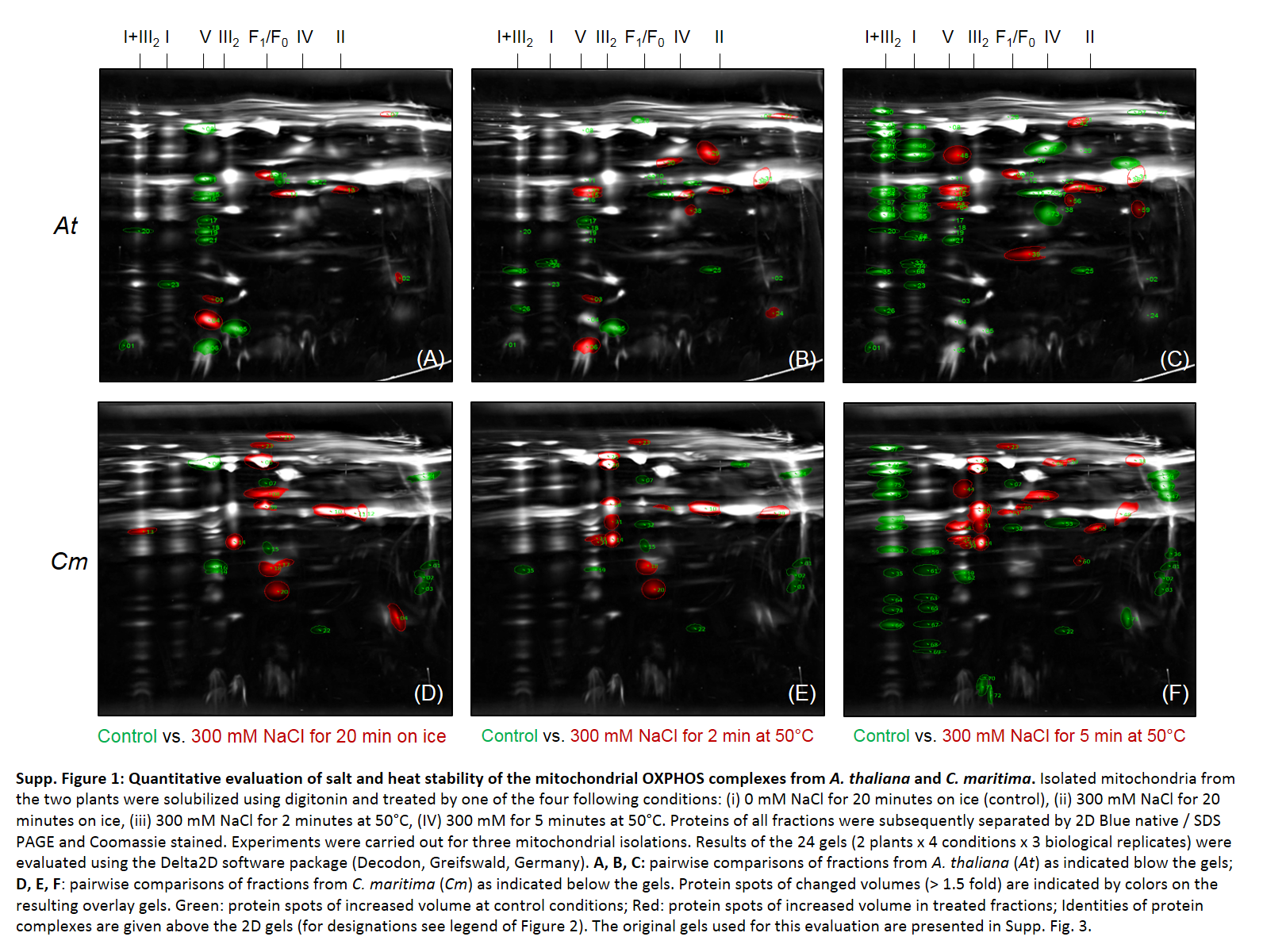

Supplement: Supplementary file 1 [file Image_1.TIF]

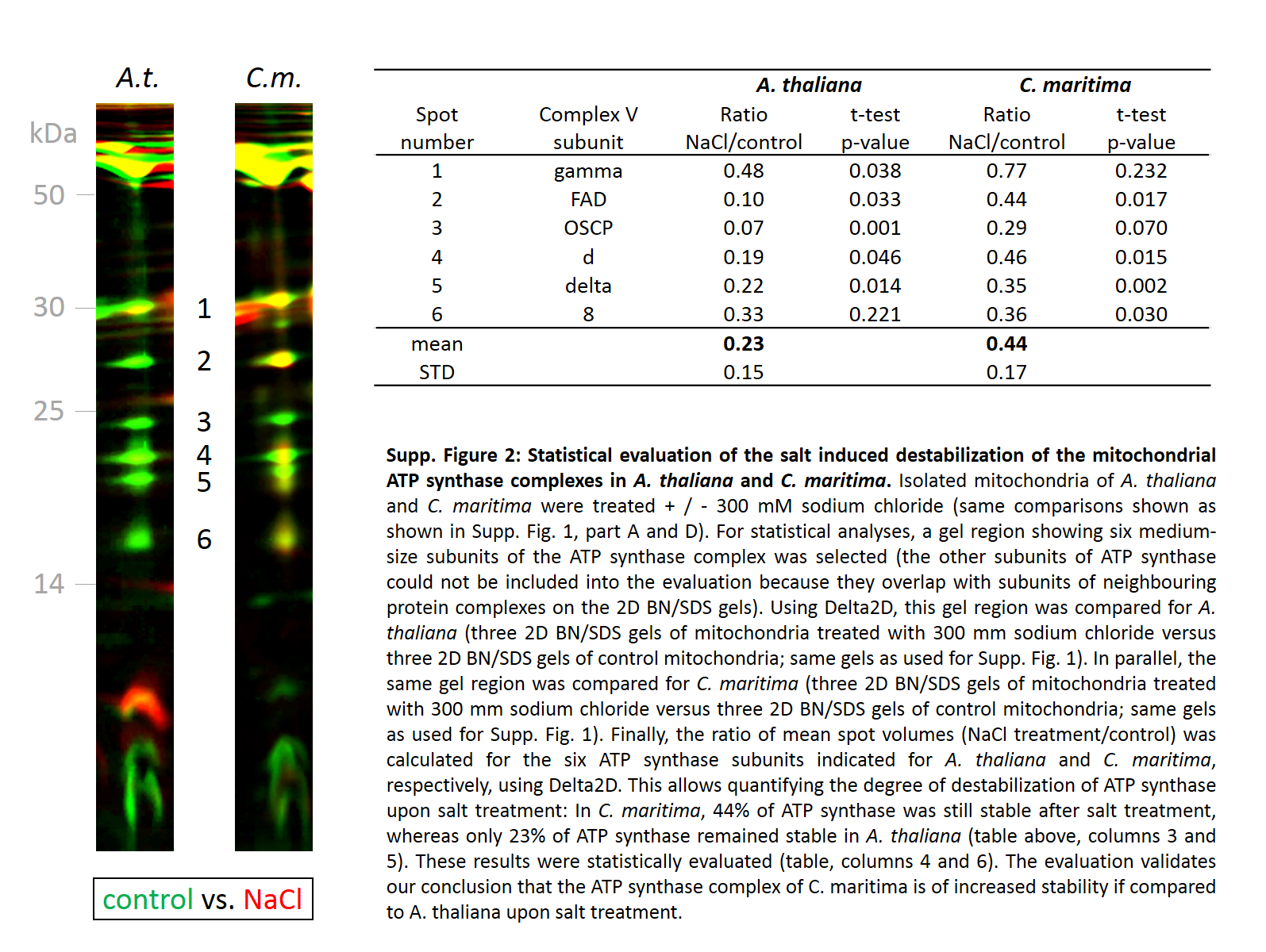

Supplement: Supplementary file 2 [file Image_2.TIF]
