# Supplementary material for: Composition and Stability of the Oxidative Phosphorylation System in the Halophile Plant Cakile maritima
Source: Front Plant Sci. 2019 Aug 13;10:1010. doi: 10.3389/fpls.2019.01010 (PMC6700300; doi:10.3389/fpls.2019.01010)
Supplement: Supplementary file 3 [file Image_3.pdf]

Arabidopsis thaliana

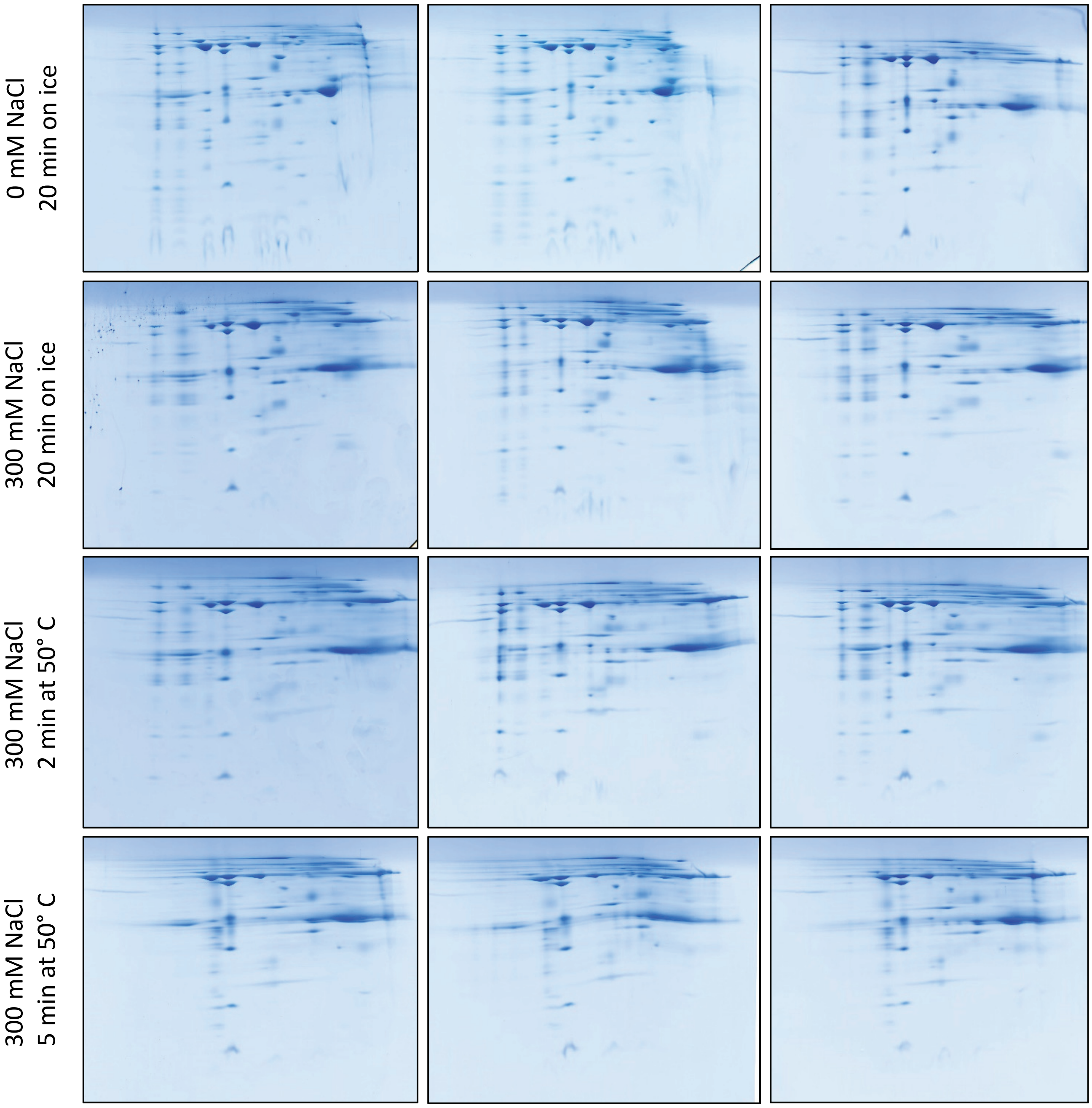

Supp. Fig. 3a: All replicates of the 2D Blue native / SDS PAGE gels used for quantitative evaluation of salt and heat stability of the mitochondrial OXPHOS complexes from *A. thaliana* (Fig. 4)

Isolated mitochondria from were solubilized using digitonin and treated by one of the four following conditions: (i) 0 mM NaCl for 20 minutes on ice (control), (ii) 300 mM NaCl for 20 minutes on ice, (iii) 300 mM NaCl for 2 minutes at 50°C, (IV) 300 mM for 5 minutes at 50°C. Proteins of all fractions were subsequently separated by 2D Blue native / SDS PAGE and Coomassie stained. Experiments were carried out for three mitochondrial isolations.

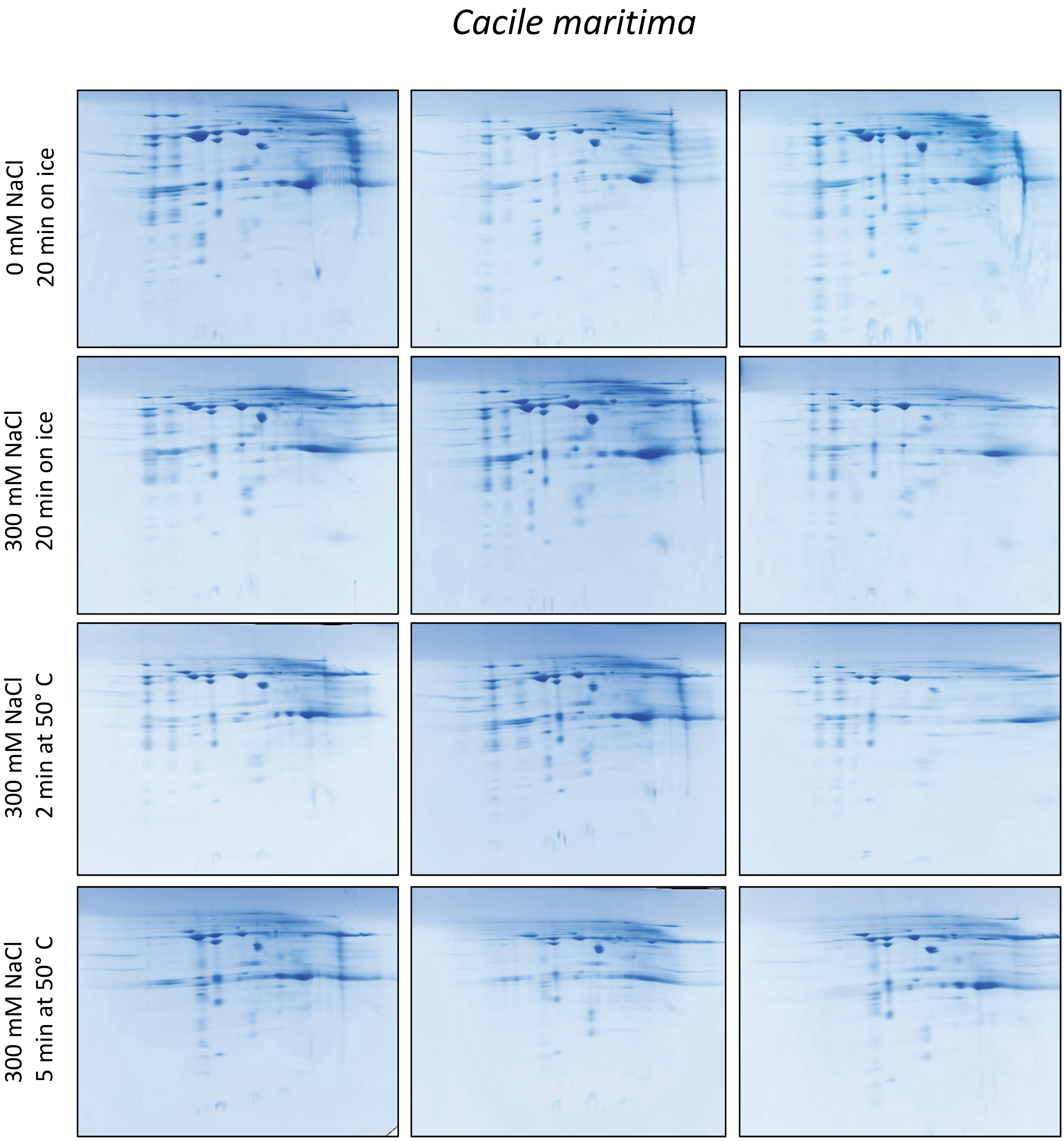

Supp. Fig. 3b: All replicates of the 2D Blue native / SDS PAGE gels used for quantitative evaluation of salt and heat stability of the mitochondrial OXPHOS complexes from *C. maritima* (Fig. 4)

Isolated mitochondria from were solubilized using digitonin and treated by one of the four following conditions: (i) 0 mM NaCl for 20 minutes on ice (control), (ii) 300 mM NaCl for 20 minutes on ice, (iii) 300 mM NaCl for 2 minutes at 50°C, (IV) 300 mM for 5 minutes at 50°C. Proteins of all fractions were subsequently separated by 2D Blue native / SDS PAGE and Coomassie stained. Experiments were carried out for three mitochondrial isolations.
